# Secondary analyses of global datasets: do obesity and physical activity explain variation in diabetes risk across populations?

**DOI:** 10.1038/s41366-021-00764-y

**Published:** 2021-02-11

**Authors:** Budour Alkaf, Alexandra I. Blakemore, Marjo-Riitta Järvelin, Nader Lessan

**Affiliations:** 1 Imperial College London Diabetes Centre, Abu Dhabi, United Arab Emirates; 2 Department of Epidemiology and Biostatistics, Imperial College London, London, UK; 3 College of Health, Medicine, and Life Sciences, Brunel University London, Uxbridge, UK; 4 Department of Medicine, Imperial College London, London, UK; 5 Centre for Life Course Health Research, Faculty of Medicine, University of Oulu, Oulu, Finland; 6 Unit of Primary Health Care and Medical Research Centre, Oulu University Hospital, Oulu, Finland

**Keywords:** Risk factors, Epidemiology

## Abstract

Type 2 diabetes rates vary significantly across geographic regions. These differences are sometimes assumed to be entirely driven by differential distribution of environmental triggers, including obesity and insufficient physical activity (IPA). In this review, we discuss data which conflicts with this supposition. We carried out a secondary analysis of publicly available data to unravel the relative contribution of obesity and IPA towards diabetes risk across different populations. We used sex-specific, age-standardized estimates from Non-Communicable Disease Risk Factor Collaboration (NCD-RisC) on diabetes (1980–2014) and obesity (1975–2016) rates, in 200 countries, and from WHO on IPA rates in 168 countries in the year 2016. NCD-RisC and WHO organized countries into nine super-regions. All analyses were region- and sex-specific. Although obesity has been increasing since 1975 in every part of the world, this was not reflected in a proportional increase in diabetes rates in several regions, including Central and Eastern Europe, and High-income western countries region. Similarly, the association of physical inactivity with diabetes is not homogeneous across regions. Countries from different regions across the world could have very similar rates of diabetes, despite falling on opposite ends of IPA rate spectrum. The combined effect of obesity and IPA on diabetes risk was analyzed at the worldwide and country level. The overall findings highlighted the larger impact of obesity on disease risk; low IPA rates do not seem to be protective of diabetes, when obesity rates are high. Despite that, some countries deviate from this overall observation. Sex differences were observed across all our analyses. Overall, data presented in this review indicate that different populations, while experiencing similar environmental shifts, are apparently differentially subject to diabetes risk. Sex-related differences observed suggest that males and females are either subject to different risk factor exposures or have different responses to them.

## Introduction

Type 2 diabetes (T2D) is a complex metabolic disorder with worryingly increasing rates in several regions in the world. The development of T2D reflects a complex interplay of adverse lifestyle exposures with potentially increased genetic predisposition. The rapid worldwide increase in T2D rates is primarily a consequence of ageing in most populations, and the increase of obesity levels and lack of physical activity [1].

The link between obesity and T2D has been recognized as early as the 1920’s [2]. Various measures of being overweight have repeatedly been shown to strongly associate with increased risk of T2D [3–6]. Similarly, the impact of physical activity on preventing and managing T2D has been established through cross-sectional and prospective studies. Overall, these studies have demonstrated the [1] independent association of physical inactivity with T2D risk, [2] association of increased sedentary behaviors with elevated T2D risk, and [3] independent-beneficial effect of moderate and high occupational physical activity in reducing T2D risk [7–15]. These associations were largely based on individuals of White ethnicity.

T2D rates vary significantly between countries, geographic regions, and ethnic groups [16]. An ethnic group is a group of people who share cultural ideas and beliefs that have been a part of their community for generations, including language, religion, history, types of food, beliefs, and celebrations. People from the same ethnic group do not always share the same religion, and on the other hand, people from different ethnic groups may share the same religion, despite being from different cultures. Southwest Asia, for example, is home to many different ethnic groups who share similar religions.

Understanding difference in T2D risk across ethnic groups has been very challenging and not straightforward. Although the differential distribution of established behavioral and environmental exposures across geographic regions and ethnic populations can account for part of this variation, there is still a big part that remains unexplained. Part of the ethnic disparity in T2D risk is thought to be due to differences in glycemic control mechanisms between ethnicities [17, 18]. For example, Indian Asians are more insulin resistant than most other ethnic groups, while T2D pathogenesis among African Americans appears to be largely driven by β-cell dysfunction, rather than by insulin resistance [19, 20].

Preferential distribution of fat storage was also shown to contribute to difference in risk of developing T2D, both within populations of the same ethnicity, and across different ethnic groups [21–23]. For a given amount of body fat, individuals with excessive intra-abdominal or visceral adipose tissue have a substantially greater risk of being insulin resistant and developing T2D [24, 25]. Whereas, higher relative distribution of subcutaneous adipose tissue plays a protective role on T2D risk [26]. Fat storage distribution differs across ethnicities. Two populations may have identical BMI distributions, but could still have large differences in intra-abdominal fat accumulation [21–23]. The Asian Indian population, for example, seems to preferentially accumulate truncal fat, which has been proposed to explain the excessive insulin resistance and high T2D prevalence in this ethnic group, despite the absence of significant obesity as assessed by BMI [19, 20].

Differences in T2D risk across populations could also be explained by differential genetic susceptibility. Although a whole population may be subject to a changed environment, genetic factors influence how each individual within a population responds to that exposure: some people carry genetic variants that increase susceptibility to obesity and/or T2D. Such genetic susceptibility factors may be more common in some ethnic groups than others, and this may contribute to the variation in T2D risk observed between different populations. However, the prevalence of genetic risk factors will not have changed during the relatively short time period under consideration, so recent differences observed in populations of the same or similar ethnicity are likely to reflect environmental exposures. In particular, there are very different rates of obesity and physical activity between different populations and these may contribute to the observed differences in T2D risk.

Several trans-ethnic studies were directed to elucidate the impact of ethnicity on the association of age, BMI, and physical inactivity with T2D. Overall, their findings confirmed large ethnic, regional- and sex-dependent differences in age-adjusted prevalence of T2D, but also showed that ethnic-specific variations were not fully explained by differences in BMI between ethnic groups [21]. In addition, they reported ethnic-specific variation in T2D risk conferred by BMI and weight gain, with the biggest detrimental effects among Asians in comparison to other ethnic groups studied [27, 28]. Cross-sectional studies also demonstrated that differences in T2D rates between ethnic groups were not explained by different levels of physical activity [29–31]. Interestingly, reduced physical activity levels were not associated with T2D risk in several ethnic groups studied. A more recent prospective study supported these findings [32].

In spite of these efforts, there is still an underrepresentation of various ethnic populations in existing studies and a lack of studies that cover a wider range of ethnic populations. Networks and organizations like the Non-Communicable Disease Risk Factor Collaboration (NCD-RisC) and World Health Organization (WHO) provide an opportunity to fill this research gap. Together, they provide worldwide rigorous and timely data on risk factors for noncommunicable diseases, including diabetes, obesity, and physical inactivity. In 2016 and 2017, NCD-RisC [33, 34] provided the lengthiest and most complete estimates of worldwide adult diabetes rates, and a complete picture of trends in mean BMI and prevalence rates of obesity for all countries in the world with the longest observation period. NCD-RisC diabetes estimates presented in this review are based on all diabetes cases worldwide, including people with type 1 diabetes (T1D) however, T2D accounts for roughly 90% of these cases. In addition to NCD-RisC data, in 2018, a group from WHO [35] provided the most complete description of global-, regional-, and country levels of insufficient physical activity, and presented regional and global trends over time.

In our secondary-analysis review, we aimed to compile publicly available estimates by NCD-RisC and WHO on diabetes, obesity, and insufficient physical activity rates and trends in adults, to unravel the relative contribution of obesity and physical inactivity on diabetes risk across different populations. We present and discuss region- and sex-specific [1] changes in obesity and diabetes trends across 35–40 years, and [2] the association of obesity and insufficient physical activity with diabetes. We also analyzed country- and sex-specific joint effects of obesity and insufficient physical activity on diabetes risk.

We have conducted a systematic review as part of this review paper. Details are presented in supplementary information (Supplementary Table 1–3).

## Materials and methods

### Data retrieval: publicly available databases for estimates of diabetes, obesity, and insufficient physical activity rates

Region-, country-, and sex-specific estimates of [1] diabetes prevalence rates from 1980 to 2014, and [2] obesity prevalence rates from 1975 to 2016, were downloaded from www.ncdrisc.org. All estimates downloaded and used in this study covered males and females aged ≥18 years. Region-, country-, and sex-specific estimates of insufficient physical activity prevalence rates in 2016 were retrieved from a recent publication by a group from WHO [35].

Estimates of diabetes, obesity, and insufficient physical activity rates were available for 200 (diabetes and obesity) and 168 (insufficient physical activity) countries, respectively. Estimates were available as age-standardized prevalence rates with upper and lower 95% uncertainty intervals, and arranged by sex, country, region, and year. NCD-RisC and WHO organized countries into 21 regions and nine super-regions (Tables 1, 2), mostly on the basis of geography and national income. The exception was a region consisting of High-income English-speaking countries: cardiometabolic risk factors, including BMI, have very similar trends in these countries, which may be distinct from other countries within their geographic region [33–35]. Region definitions by NCD-RisC and WHO were equivalent. Region-specific analyses here were based on the nine super-regions, namely [1] Central Asia, Middle East and North Africa, [2] Central and Eastern Europe, [3] East and South East Asia, [4] High-income Asia Pacific, [5] High-income Western countries, [6] Latin America and Caribbean, [7] Oceania, [8] South Asia, and [9] Sub-Saharan Africa.

**Table 1:** List of countries included in NCD-RisC analyses to estimate diabetes and obesity prevalence rates, by super-region and region.

Super-region	Region	Number of countries	Countries
Sub-Saharan Africa	Central Africa	6	Angola, Central African Republic, Congo, DR Congo, Equatorial Guinea, Gabon
	East Africa	17	Burundi, Comoros, Djibouti, Eritrea, Ethiopia, Kenya, Madagascar, Malawi, Mauritius, Mozambique, Rwanda, Seychelles, Somalia, Sudan (former), Tanzania, Uganda, Zambia
	Southern Africa	6	Botswana, Lesotho, Namibia, South Africa, Swaziland, Zimbabwe
	West Africa	19	Benin, Burkina Faso, Cabo Verde, Cameroon, Chad, Cote d’Ivoire, Gambia, Ghana, Guinea, Guinea Bissau, Liberia, Mali, Mauritania, Niger, Nigeria, Sao Tome and Principe, Senegal, Sierra Leone, Togo
Central Asia, Middle East and North Africa	Central Asia	9	Armenia, Azerbaijan, Georgia, Kazakhstan, Kyrgyzstan, Mongolia, Tajikistan, Turkmenistan, Uzbekistan
	Middle East and North Africa	19	Algeria, Bahrain, Egypt, Iran, Iraq, Jordan, Kuwait, Lebanon, Libya, Morocco, Occupied Palestinian Territory, Oman, Qatar, Saudi Arabia, Syrian Arab Republic, Tunisia, Turkey, United Arab Emirates, Yemen
South Asia	South Asia	6	Afghanistan, Bangladesh, Bhutan, India, Nepal, Pakistan
East and South East Asia	East Asia	4	China, China (Hong Kong SAR), North Korea, Taiwan
	South East Asia	12	Brunei Darussalam, Cambodia, Indonesia, Lao PDR, Malaysia, Maldives, Myanmar, Philippines, Sri Lanka, Thailand, Timor-Leste, Viet Nam
Oceania	Polynesia and Micronesia	13	American Samoa, Cook Islands, French Polynesia, Kiribati, Marshall Islands, Micronesia (Federated States of), Nauru, Niue, Palau, Samoa, Tokelau, Tonga, Tuvalu
	Melanesia	4	Fiji, Papua New Guinea, Solomon Islands, Vanuatu
High-income Asia Pacific	High-income Asia Pacific	3	Japan, Singapore, South Korea
Latin America and Caribbean	Andean Latin America	3	Bolivia, Ecuador, Peru
	Caribbean	18	Antigua and Barbuda, Bahamas, Barbados, Belize, Bermuda, Cuba, Dominica, Dominican Republic, Grenada, Guyana, Haiti, Jamaica, Puerto Rico, Saint Kitts and Nevis, Saint Lucia, Saint Vincent and the Grenadines, Suriname, Trinidad and Tobago
	Central Latin America	9	Colombia, Costa Rica, El Salvador, Guatemala, Honduras, Mexico, Nicaragua, Panama, Venezuela
	Southern Latin America	5	Argentina, Brazil, Chile, Paraguay, Uruguay
High-income Western countries	High-income English-speaking countries	6	Australia, Canada, Ireland, New Zealand, United Kingdom, United States of America
	North Western Europe	12	Austria, Belgium, Denmark, Finland, Germany, Greenland, Iceland, Luxembourg, Netherlands, Norway, Sweden, Switzerland
	South Western Europe	9	Andorra, Cyprus, France, Greece, Israel, Italy, Malta, Portugal, Spain
Central and Eastern Europe	Central Europe	13	Albania, Bosnia and Herzegovina, Bulgaria, Croatia, Czech Republic, Hungary, Macedonia (TFYR), Montenegro, Poland, Romania, Serbia, Slovakia, Slovenia
	Eastern Europe	7	Belarus, Estonia, Latvia, Lithuania, Moldova, Russian Federation, Ukraine

**Table 2:** List of countries included in the analysis performed in Guthold R paper, by super-region.

Super-region	Number of countries	Countries
Central Asia, Middle East and North Africa	23	Algeria, Armenia, Egypt, Georgia, Iran (Islamic Republic of), Iraq, Jordan, Kazakhstan, Kuwait, Kyrgyzstan, Lebanon, Libya, Mongolia, Morocco, Oman, Qatar, Saudi Arabia, State of Palestine, Tajikistan, Tunisia, Turkey, United Arab Emirates, Uzbekistan
Central and Eastern Europe	17	Belarus, Bosnia and Herzegovina, Bulgaria, Croatia, Czech Republic, Estonia, Hungary, Latvia, Lithuania, Poland, Republic of Moldova, Romania, Russian Federation, Serbia, Slovakia, Slovenia, Ukraine
East and South East Asia	13	Brunei Darussalam, Cambodia, China, Indonesia, Lao People’s Democratic Republic, Malaysia, Maldives, Myanmar, Philippines, Sri Lanka, Thailand, Timor-Leste, Viet Nam
High-income Asia Pacific	3	Japan, Republic of Korea, Singapore
High-income Western countries	24	Andorra, Australia, Austria, Belgium, Canada, Cyprus, Denmark, Finland, France, Germany, Greece, Ireland, Italy, Luxembourg, Malta, Netherlands, New Zealand, Norway, Portugal, Spain, Sweden, Switzerland, United Kingdom, United States of America
Latin America and Caribbean	25	Argentina, Bahamas, Barbados, Bermuda, Brazil, British Virgin Islands, Cayman Islands, Chile, Colombia, Costa Rica, Cuba, Dominica, Dominican Republic, Ecuador, Grenada, Guatemala, Jamaica, Mexico, Paraguay, Saint Kitts and Nevis, Saint Lucia, Suriname, Trinidad and Tobago, Uruguay, Venezuela (Bolivarian Republic of)
Oceania	17	American Samoa, Cook Islands, Fiji, French Polynesia, Kiribati, Marshall Islands, Micronesia (Federated States of), Nauru, Niue, Palau, Papua New Guinea, Samoa, Solomon Islands, Tokelau, Tonga, Tuvalu, Vanuatu
South Asia	5	Bangladesh, Bhutan, India, Nepal, Pakistan
Sub-Saharan Africa	41	Benin, Botswana, Burkina Faso, Cabo Verde, Cameroon, Central African Republic, Chad, Comoros, Congo, Côte d’Ivoire, Democratic Republic of the Congo, Eritrea, Ethiopia, Gabon, Gambia, Ghana, Guinea, Kenya, Lesotho, Liberia, Madagascar, Malawi, Mali, Mauritania, Mauritius, Mozambique, Namibia, Niger, Nigeria, Rwanda, São Tomé and Principe, Senegal, Seychelles, Sierra Leone, South Africa, Swaziland, Togo, Uganda, United Republic of Tanzania, Zambia, Zimbabwe

### Description of Non-communicable Disease Risk Factor Collaboration (NCD-RisC) data sources

NCD-RisC is a worldwide network of health researchers and practitioners who aim to document systematically worldwide trends and variations in noncommunicable diseases (NCDs). Here we used yearly data for diabetes and obesity prevalence rates.

Diabetes was defined as fasting plasma glucose of 7.0 mmol/L or higher, history of diagnosis with diabetes, or use of insulin or oral hypoglycemic drugs [33]. To estimate diabetes prevalence rates, NCD-RisC identified, assessed, and reanalyzed population-based health-examination surveys that had measured at least one diabetes biomarker, and then converted diabetes prevalence in sources that had defined diabetes through 2hOGTT or HbA1c or used cutoff other than 7.0 mmol/L for fasting plasma glucose, to a corresponding prevalence based on the definition above [33]. Data sources included were representative of a national, subnational, or community population. Data sources that relied entirely on self-reported history of diagnosis were excluded. More detailed methods for identifying and accessing data sources were described in here [33].

Obesity was defined as BMI ≥ 30 kg/m^2^. To estimate obesity prevalence rates in adults, NCD-RisC pooled and analyzed population-based studies that measured height and weight in adults aged 20 years and older [34]. They included data collected on samples of a national, subnational, or community populations, which had measured weight and height. Data sources that were based on self-reported weight and height were excluded, due to being subject to biases that vary by geography, time, age, sex, and socioeconomic characteristics [34].

The statistical models used by NCD-RisC to estimate prevalence by country, year, sex, and age are described in detail in their statistical paper and related substantive papers [33, 34, 36–38].

### Description of World Health Organization (WHO) data sources

WHO estimated prevalence rates of insufficient physical activity in adults aged 18 years and older in 168 countries for three World Bank income groups, nine regions (Table 2), and globally from 2001 to 2016. Data were published in Lancet Global Health journal in 2018 [35]. Insufficient physical activity was defined as adults not meeting the WHO recommendations on physical activity for health—i.e., at least 150 min of moderate-intensity, or 75 min of vigorous-intensity physical activity per week, or any equivalent combination of the two.

Data sources included fulfilled the following criteria: [1] the survey questionnaire explicitly included physical activity across four key domains—i.e., work in the household (paid or unpaid), transport to get to and from places (i.e., walking and cycling), leisure time activities (i.e., sports and active recreation); [2] data were collected through random sampling with a sample size of at least 200, and were representative of a national or defined subnational population[3]; prevalence of insufficient physical activity was reported by age and sex, according to current WHO [39], or former physical activity recommendations [35, 40]. Physical activity data collected using wearable devices were not included due to limited comparability with self-reported data.

The statistical models used by WHO to derive global and regional estimates were described in detail in their paper [35].

### Current data analysis approaches

The following describes the current data analysis approaches used in this secondary-analysis review. Region- and sex-specific differences in diabetes prevalence rates and trends were assessed using scatter plots to compare the distribution of diabetes prevalence rates in males against that in females across 200 countries. This was done separately for the year 1980 and 2014, and for the change in diabetes prevalence throughout the 34 years (change in diabetes prevalence throughout the 34 years (1980–2014) studied = diabetes prevalence in 2014 − diabetes prevalence in 1980). These analyses were repeated to assess region- and sex-specific differences in obesity prevalence rates in 1975 and 2016, and the change in obesity prevalence throughout the 41 years (1975–2016) studied (change in obesity prevalence throughout the 41 years = obesity prevalence in 2016 − obesity prevalence in 1975). Region- and sex-specific correlation analyses of obesity with diabetes were based on obesity and diabetes prevalence rates in 200 countries in years 2016 and 2014, respectively. Region- and sex-specific correlation analyses of insufficient physical activity with diabetes were based on insufficient physical activity and diabetes prevalence rates in 163 countries in the year 2016 and 2014, respectively. Shapiro-Wild tests were performed to test for the normality of diabetes, obesity, and insufficient physical activity prevalence rate distribution. Correlation was tested using Pearson Correlation test. Significance was set at *p* < 0.05. Country- and sex-specific joint effects of obesity and insufficient physical activity prevalence rates with diabetes were based on estimates in 163 countries in years 2014 (diabetes) and 2016 (obesity and insufficient physical activity). Estimate of obesity, insufficient physical activity, and diabetes rates were each split into three tertiles. For each of obesity and insufficient physical activity rates, countries that fell in the first, second, and third tertiles were coded as low, moderate, and high, respectively. Diabetes risk was defined on the basis of the tertile split, i.e., countries that fell in the first tertile (with the lowest rates of diabetes) were coded as low risk, and those that fell in the second and third tertiles, were coded as moderate and high risk, respectively. All analyses were performed in Prism (Version 8).

## Results

### Region- and sex-specific trends in diabetes rates from 1980 to 2014

**Fig. 1:**
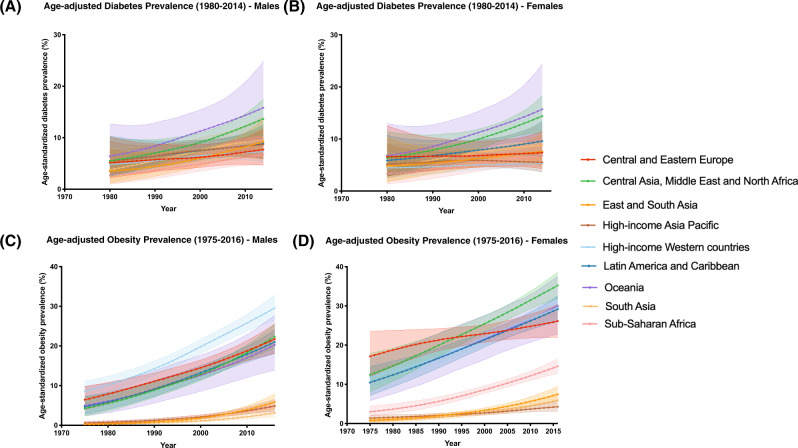
Region and sex-specifc trends of diabetes and obesity in the last 35-40 years. **A** Age-adjusted diabetes prevalence (1980–2014) in males, **B** Age-adjusted diabetes prevalence (1980–2014) in females, **C** Age-adjusted obesity prevalence (1975–2016) in males, and **D** Age-adjusted obesity prevalence (1975–2016) in females. Age-standardized diabetes and obesity prevalence rates as estimated by Non-Communicable Disease risk Collaboration (NCD-RisC) group, for each year from 1980 to 2014, and 1975 to 2016, respectively. NCD-RisC group reported diabetes and obesity prevalence estimates in 200 countries, which were categorized into nine super-regions, including Central and Eastern Europe, Central Asia Middle East and North Africa, East and South East Asia, High-income Asia Pacific, High-income Western countries, Latin America and Caribbean, Oceania, South Asia, and Sub-Saharan Africa.

Based on our analysis of NCD-RisC data, in 2014, age-standardized diabetes prevalence was high in Oceania among both males (15.8%) and females (14.4%) (Fig. 1A, B). During the 34 years studied (1980–2014), Oceania and Central Asia, Middle East and North Africa regions were estimated to have the highest rates of diabetes across all years, particularly after 1985 (Fig. 1A, B). Sex-specific differences in diabetes prevalence increased with the years; in 1980, overall diabetes rates were almost identical across sexes (Supplementary Figure 1A). By 2014, diabetes rates were higher around the world, rising more rapidly in some countries/regions than others. Overall, increases in diabetes prevalence were higher in males compared to females (Supplementary Figure 1C). In both males and females, during the 34 years (1980–2014), the largest increase in diabetes prevalence was in Oceania and Central Asia, Middle East and North Africa, whereas the lowest increase in diabetes prevalence was in the High-Income Western countries region. There were sex differences between populations too: rates were higher in males, in High-income Western countries, and higher in females among countries in Latin America and Caribbean (Supplementary Figure 1B).

### Region- and sex-specific trends in obesity rates from 1975 to 2016

Throughout the 41 years studied (1975–2016), obesity rates in males were high in High-income Western countries, and lowest in South Asia, particularly post-2000 (Fig. 1C). In females, obesity rates were high in Central and Eastern Europe in the first 15 years, but this increasing trend slowed after 1990 (Fig. 1D). Between 1990 and 2016, obesity rates in females were high in Central Asia Middle East and North Africa region, and low in High-income Asia Pacific region, particularly in the last 5 years (2010–2016) (Fig. 1D). Overall, females had higher rates of obesity compared to males across all regions both in 1975 and 2016 (Supplementary Figure 2A, B). In both males and females, during the 41 years (1975–2016), the largest increase in obesity prevalence was in Oceania, whereas the lowest increase in obesity prevalence was in South Asia and High-income Asia Pacific regions (Supplementary Figure 2C).

### Contribution of obesity towards explaining differences in diabetes risk across populations

In order to assess region- and sex-specific correlation of obesity with diabetes, we used NCD-RisC estimates of diabetes prevalence rates in 2014 and obesity prevalence rates in 2016 for 200 countries in nine regions. In males, obesity rates were significantly correlated with diabetes rates in only four regions, with the strongest correlation in Oceania, followed by Central Asia Middle East and North Africa, East and South East Asia, and Sub-Saharan Africa (Table 3). Whereas in females, a significant correlation was observed across all regions, except for Central and Eastern Europe, and High-income Asia Pacific. As in males, the strongest correlation was observed in Oceania and Central Asia Middle East and North Africa regions (Table 3).

**Table 3:** Pearson correlation test for region-specific correlation of obesity and insufficient physical activity (IPA) with diabetes.

Males	Obesity		IPA	
Region	R-squared	*P* value^†^	R-squared	*P* value^†^
Central and Eastern Europe	0.019	0.567	0.007	0.765
Central Asia Middle East and North Africa	0.747	<0.0001	0.486	0.0002
East and South East Asia	0.491	0.0025	0.281	0.062
High-income Asia Pacific	0.098	0.797	Too few pairs (only two countries)	
High-income Western Countries	0.134	0.06	0.243	0.014
Latin America and Caribbean	0.101	0.064	0.077	0.2
Oceania	0.827	<0.0001	0.134	0.015
South Asia	0.504	0.114	0.042	0.74
Sub-Saharan Africa	0.36	<0.0001	0.137	0.019

Females				
Region	R-squared	*P* value^†^	R-squared	*P* value^†^
Central and Eastern Europe	0.045	0.372	0.671	0.0001
Central Asia Middle East and North Africa	0.669	<0.0001	0.396	0.0013
East and South East Asia	0.619	0.0003	0.105	0.281
High-income Asia Pacific	0.575	0.452	Too few pairs (only two countries)	
High-income Western Countries	0.593	<0.0001	0.348	0.0024
Latin America and Caribbean	0.347	0.0002	0.003	0.816
Oceania	0.7	<0.0001	0.114	0.184
South Asia	0.737	0.029	0	0.96
Sub-Saharan Africa	0.55	<0.0001	0.106	0.041

*IPA* Insufficient physical activity.

^†^*P* value not corrected for multiple testing.

**Fig. 2:**
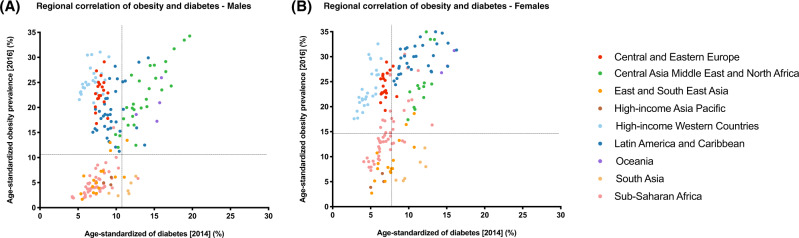
Region- and sex-specific correlation of obesity with diabetes. **A** Males and **B** Females. The data presented was based on Non-Communicable Disease risk Collaboration (NCD-RisC) group estimates of diabetes and obesity, in 2014 and 2016, respectively. Scattergraphs A and B represent the age-standardized prevalence rates of diabetes in 2014 (*x*-axis) against obesity in 2016 (*y*-axis), across 200 countries, in males and females, respectively. For each graph, the vertical dotted line represents the worldwide age-standardized prevalence rate of diabetes in 2014, and the horizontal line represents the worldwide age-standardized prevalence rate of obesity in 2016. The 200 countries were categorized into nine super-regions and color-coded in the figure, which include Central and Eastern Europe, Central Asia Middle East and North Africa, East and South East Asia, High-income Asia Pacific, High-income Western countries, Latin America and Caribbean, Oceania, South Asia, and Sub-Saharan Africa.

In line with these observations, a majority of countries with obesity prevalence rates lower than the worldwide mean (10.8% in males, 14.9% in females), also had lower diabetes prevalence rates compared to the worldwide mean (9% in males, 7.9% in females), and vice versa. This was observed in males and females (Fig. 2). Despite this well-established association, several countries, particularly in Latin America and Caribbean and Central and Eastern Europe regions yield surprising data: they have some of the highest rates of obesity, but still have some of the lowest rates of diabetes. When comparing diabetes and obesity rate trends between 1980 and 2016 (Supplementary Figure 3), the increasing obesity trends in some regions, like Central Asia, Middle East and North Africa reflect the increasing diabetes trend in the region. In other regions, however, including Central Eastern Europe and High-income Western countries regions, diabetes rates have been consistent during that time period, despite the increasing trends of obesity in these regions.

### Contribution of insufficient physical activity towards explaining differences in diabetes risk across populations

To assess region- and sex-specific correlation of insufficient physical activity with diabetes, we extracted estimates from year 2016 of insufficient physical activity rates for 163 countries in nine regions, as reported by Guthold et al. [35]. Diabetes prevalence rates were based on NCD-RisC estimates in 2014. Prevalence rates of insufficient physical activity were higher in females compared to males across all regions (Supplementary Figure 4). In males, insufficient physical activity rates were significantly correlated with diabetes rates in only three regions, with the strongest correlation in Central Asia Middle East and North Africa, followed by High-income Western countries and Sub-Saharan Africa (Table 3). Whereas in females, the correlation of insufficient physical activity rates with diabetes rates was only significant in four regions, with the strongest correlation in Central and Eastern Europe, followed by Central Asia Middle East and North Africa, High-income Western countries, and Sub-Saharan Africa (Table 3).

**Fig. 3:**
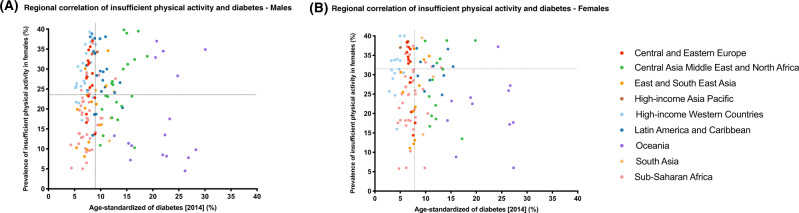
Region- and sex-specific correlation of insufficient physical activity with diabetes. **A** Males and **B** Females. The data presented was based on diabetes prevalence rates estimates by Non-Communicable Disease risk Collaboration (NCD-RisC) group in 2014, and insufficient physical activity prevalence rates estimates by World Health Organization (WHO) in 2016. Scattergraphs A and B represent the age-standardized prevalence rates of diabetes in 2014 (*x*-axis) against insufficient physical activity 2016 (*y*-axis), across 163 countries, in males and females, respectively. For each graph, the vertical dotted line represents the worldwide age-standardized prevalence rate of diabetes in 2014, and the horizontal line represents the worldwide age-standardized prevalence rate of insufficient physical activity in 2016. The 163 countries were categorized into nine super-regions and color-coded in the figure, which include Central and Eastern Europe, Central Asia Middle East and North Africa, East and South East Asia, High-income Asia Pacific, High-income Western countries, Latin America and Caribbean, Oceania, South Asia, and Sub-Saharan Africa.

Figure 3 demonstrates the correlation of insufficient physical activity with diabetes across all nine regions in males (Fig. 3A) and females (Fig. 3B). A majority of countries with insufficient physical activity rates lower than the worldwide mean (23.4% in males, 31.7% in females) also had lower diabetes rates compared to the worldwide mean (9% in males, 7.9% in females), and vice versa. This was observed in males and females (Fig. 3). Despite that, several countries, particularly in High-income Western countries and Central and Eastern Europe regions, have some of the highest rates of insufficient physical activity, but still have some of the lowest rates of diabetes. Other countries, in Oceania in particular, have some of the lowest rates of insufficient physical activity despite having some of the highest rates of diabetes in the world.

### Joint effects of obesity and physical inactivity levels with diabetes

**Fig. 4:**
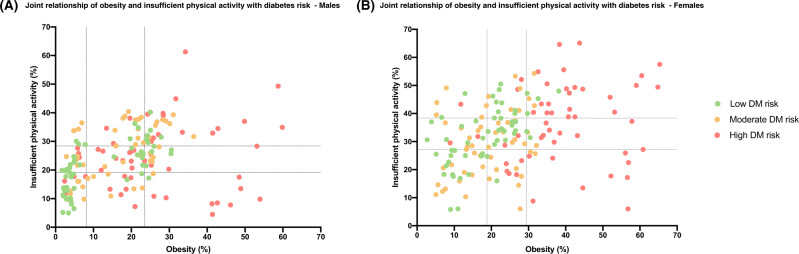
Joint effects of obesity and insufficient physical activity levels with diabetes risk. **A** Males and **B** Females. The diabetes and obesity rates were estimated by Non-Communicable Disease Risk Collaboration (NCD-RisC) group in 2014 and 2016, respectively. Insufficient physical activity rates were estimated by World Health Organization (WHO) in 2016. Scattergraphs represent distribution of age-standardized prevalence rates of obesity (*x*-axis) against insufficient physical activity rates in 163 countries. Estimates of obesity, insufficient physical actiivty, and diabetes were grouped into tertiles. For diabetes rates, countries that fell in the first tertile (with the lowest rates of diabetes) were coded as low risk (green), and those that fell in the second and third tertiles, were coded as moderate (yellow) and high (red) risk, respectively. Dotted vertical and horizontal lines represent the first and second tertiles of obesity and insufficient physical activity rates, respectively.

In order to assess country- and sex-specific joint effects of obesity and physical inactivity on diabetes, we analyzed the distribution of obesity and insufficient physical activity rates in 163 countries in relation to diabetes risk (Fig. 4). Overall, males and females across all countries have varying risk of diabetes (low, moderate, or high), irrespective of differences in their insufficient physical activity levels. Also, countries with obesity rates above 30% have high diabetes risk, irrespective of their insufficient physical activity rates. However, there is no such clear cutoff for insufficient physical activity (Fig. 4).

In spite of this overall picture, there are some countries that are outliers of this observation, and there seems to be a difference between sexes. A majority of males with high obesity rates have a high risk of diabetes; however, there are males in various countries with high diabetes risk despite having low or moderate rates of obesity. That is not the case in females, where the majority of countries with high obesity rates also have high risk of diabetes (Fig. 4). In addition, males but not females, in some countries of South Asia, including Bhutan, Bangladesh, and Nepal, have high diabetes risk despite having low rates of both obesity and insufficient physical activity (Table 4). In contrast to this scenario, males and females in some countries, particularly in High-income Western region, have a relatively low risk of diabetes despite having high rates of both obesity and insufficient physical activity (Table 4).

**Table 4:** Countries that fall as outliers of the positive association of obesity and insufficient physical activity (IPA) with diabetes risk in males and females.

Country	Region	Obesity	IPA	Diabetes
Males				
Bhutan	South Asia	Low	Low	High
Bangladesh	South Asia	Low	Low	High
Nepal	South Asia	Low	Low	High
Seychelles	Sub-Saharan Africa	Low	Low	High
Cabo Verde	Sub-Saharan Africa	Low	Low	Moderate
Comoros	Sub-Saharan Africa	Low	Low	Moderate
Gambia	Sub-Saharan Africa	Low	Low	Moderate
China	East and South East Asia	Low	Low	Moderate
Lao PDR	East and South East Asia	Low	Low	Moderate
Germany	High-income Western Countries	High	High	Low
Greece	High-income Western Countries	High	High	Low
Belgium	High-income Western Countries	High	High	Low
Norway	High-income Western Countries	High	High	Low
Ireland	High-income Western Countries	High	High	Low
United Kingdom	High-income Western Countries	High	High	Low
Romania	Central and Eastern Europe	High	High	Low
Females				
Nepal	South Asia	Low	Low	Moderate
Togo	Sub-Saharan Africa	Low	Low	Moderate
Cambodia	East and South East Asia	Low	Low	Moderate
China	East and South East Asia	Low	Low	Moderate
Myanmar	East and South East Asia	Low	Low	Moderate
Malta	High-income Western Countries	High	High	Low
United Kingdom	High-income Western Countries	High	High	Low
United States	High-income Western Countries	High	High	Low
New Zealand	High-income Western Countries	High	High	Low

Age-standardized prevalence rates of obesity, IPA, and diabetes rates in 163 countries were each split into tertiles. For each of obesity and insufficient physical activity rates, countries that fell in the first, second, and third tertiles were color-coded as low (green), moderate (yellow), and high (red), respectively. For diabetes rates, countries that fell in the first tertile (with the lowest rates of diabetes) were color-coded as low risk (green), and those that fell in the second and third tertiles, were color-coded as moderate (yellow) and high risk (red), respectively. Code for interpreting the table: Bhutan in South Asia has low obesity (green) and low IPA (green), but high diabetes risk (red).

IPA, Insufficient physical activity.

## Discussion

The rapid increase of diabetes across the world, particularly in Westernized populations, has been associated with environmental changes related to obesity. Excessive caloric intake and reduced physical activity are the main features of a high-risk environment for diabetes development, and the relationship of these environmental stressors with disease risk was sometimes considered to be straightforward. Here, we present and discuss data which conflicts with this supposition. Although obesity has been increasing since 1980 in every part of the world, this was not reflected in a proportional increase in diabetes rates in several regions, including Europe and High-income Western Countries. These data suggest that certain populations, such as those in the Middle East, are exposed to similar environmental stressors to some Westernized populations, yet have much higher rates of diabetes. Our findings, however, must be interpreted with caution. Obesity rates reported here are based on the universal BMI cut-off point of ≥30 kg/m^2^, and that could have resulted in underestimating obesity levels in some regions, and unreliable comparisons with other regions.

BMI is a simple and noninvasive method for assessing obesity and excessive fat stores within a single population, but less reliable when comparing adiposity measures between populations [41, 42]. One of the largest studies directed at the elucidation of the impact of ethnicity on the association of age and BMI with T2D was conducted by the Diabetes Epidemiology Collaborative analysis of Diagnostic criteria in Europe-Diabetes Epidemiology Collaborative analysis of Diagnostic criteria in Asia (DECODE-DECODA) study [21]. Nakagami et al. [21] analyzed studies/surveys representing different ethnic groups, including European, Maltese, Indian, Chinese, and Japanese populations. They found that the variation in T2D prevalence across ethnic groups cannot be explained by differences in BMI between populations alone, and that ethnicity modified the effect of BMI on age-adjusted prevalence of T2D [21]. These findings support the need for region- and ethnic-specific definitions of obesity.

The heterogeneity in the relationship between obesity (as measured by BMI) and diabetes observed here could also be explained by differences in the contribution of reduced insulin secretion and reduced insulin sensitivity to the development of diabetes between various populations. Patterns of fat distribution differ between ethnic populations [19, 43–45], and, thus, two populations could have identical BMI distributions, but still show large differences with respect to the accumulation of intra-abdominal fat. South Asian people, for example, are more insulin resistant and more prone to abdominal obesity and low muscle mass than other ethnic groups [19, 43–45]. This suggests that excessive insulin resistance may be related to intra-abdominal fat deposition and low muscle mass among South Asians: this could explain their increased risk of developing diabetes, despite the absence of significant obesity as assessed by BMI. This is in accordance with the data analyzed here. Obesity and physical inactivity in South Asia did not correlate with diabetes in the region overall: countries including Bhutan, Bangladesh, and Nepal were estimated to have some of the highest diabetes rates in the world despite their low obesity and activity rates.

Sex differences were observed across all our analyses. Previous studies, largely based on Europeans, showed that diabetes is more frequently diagnosed at a lower age and BMI in males, despite the fact that obesity is more common in females [46]. This trend was also observed here, not only in European populations (Central and Eastern Europe and High-income Western countries regions), but across all regions. Diversities in culture, lifestyle, and socioeconomic status are some of the factors that may impact differences between males and females in predisposition, development, and clinical presentation of diabetes [46]. Besides obesity, other risk factors, including genetic effects and epigenetic mechanisms, nutritional factors, and sedentary behaviors affect disease risk and complications differently in both sexes [46]. Indeed, in this study, females had higher insufficient physical activity rates compared to males, yet they still had similar rates of diabetes.

The secondary analyses presented here were based on two of the most well-established risk factors for T2D and their contribution to differences in disease risk across populations. It is important to note, however, that the development of T2D involves a complex interplay of adverse lifestyle and genetic exposures. This means that other factors could be confounding our findings, particularly those that are also associated with cultural and faith/religious groups: smoking, alcohol consumption, and nutrition, for example. WHO produces yearly estimates (2007–2018) of cigarette and tobacco smoking prevalence rates in more than 190 countries. Secondary analyses that account for the effect of factors like smoking, diet and nutrition, and socioeconomic status on our findings are urgently required.

Current efforts are largely focused on reducing the global health and economic burden of diabetes and on developing strategies to help prevent this “epidemic” [47–51]. These have provided guidelines as to who should be targeted in the preventive process. Our data showing that there are differences across sexes and different populations on the consequences of obesity and physical inactivity suggest that target groups for intervention strategies may have to be defined for each sex and ethnic group separately, and future studies should be aimed at defining these high risk groups.

## Supplementary information

Supplementary Figure 1

Supplementary Figure 2

Supplementary Figure 3

Supplementary Figure 4

Supplementary Table 1

Supplementary Table 2

Supplementary Table 3
