# Supplementary material for: Secondary analyses of global datasets: do obesity and physical activity explain variation in diabetes risk across populations?
Source: Int J Obes (Lond). 2021 Feb 11;45(5):944–56. doi: 10.1038/s41366-021-00764-y (PMC8081659; doi:10.1038/s41366-021-00764-y)
Supplement: Supplementary file 2 — Supplementary Figure 2 [file 41366_2021_764_MOESM2_ESM.pdf]

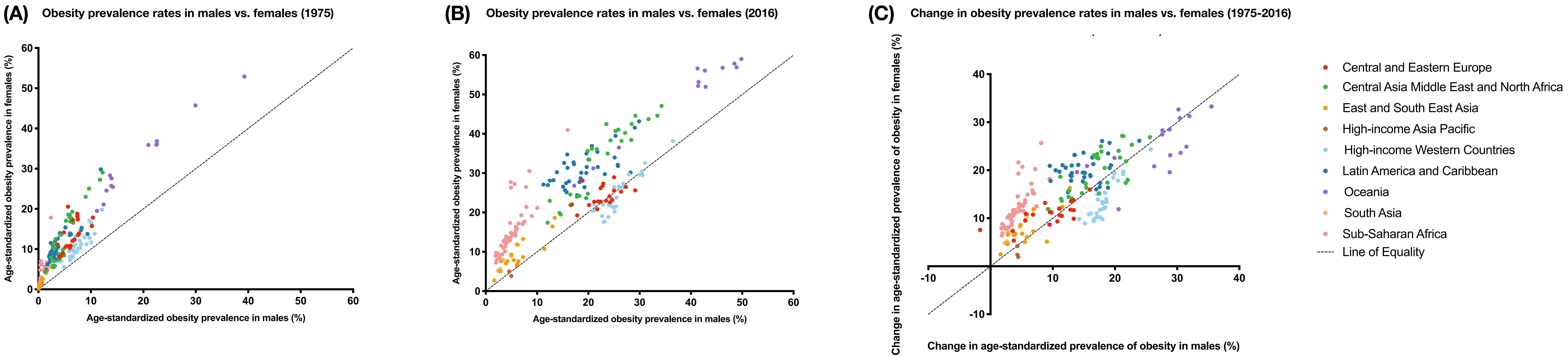

Supplementary Figure 2. Comparison of age-standardized obesity prevalence rates in (A)1975 and (B) 2016, and (C) change in obesity prevalence between 1975 and 2016 by sex. Data presented is based on Non-Communicable Disease risk Collaboration (NCD-RisC) group estimates of obesity prevalence rates in 1975 and 2016, across 200 countries. Scattergraphs A and B represent the age-standardized obesity prevalence rates in 200 countries, in males (x-axis) against females (y-axis), in 1975 and 2016, respectively. Scattergraph C represents the change in obesity prevalence rates during the 41 years (1975-2016) in 200 countries, in males (x-axis) against females (y-axis). The 200 countries were categorized into 9 regions and color coded in the figure, which include; Central and Eastern Europe, Central Asia Middle East and North Africa, East and South East Asia, High-income Asia Pacific, High-income Western countries, Latin America and Caribbean, Oceania, South Asia, and Sub-Saharan Africa.
