# Supplementary material for: Secondary analyses of global datasets: do obesity and physical activity explain variation in diabetes risk across populations?
Source: Int J Obes (Lond). 2021 Feb 11;45(5):944–56. doi: 10.1038/s41366-021-00764-y (PMC8081659; doi:10.1038/s41366-021-00764-y)
Supplement: Supplementary file 3 — Supplementary Figure 3 [file 41366_2021_764_MOESM3_ESM.pdf]

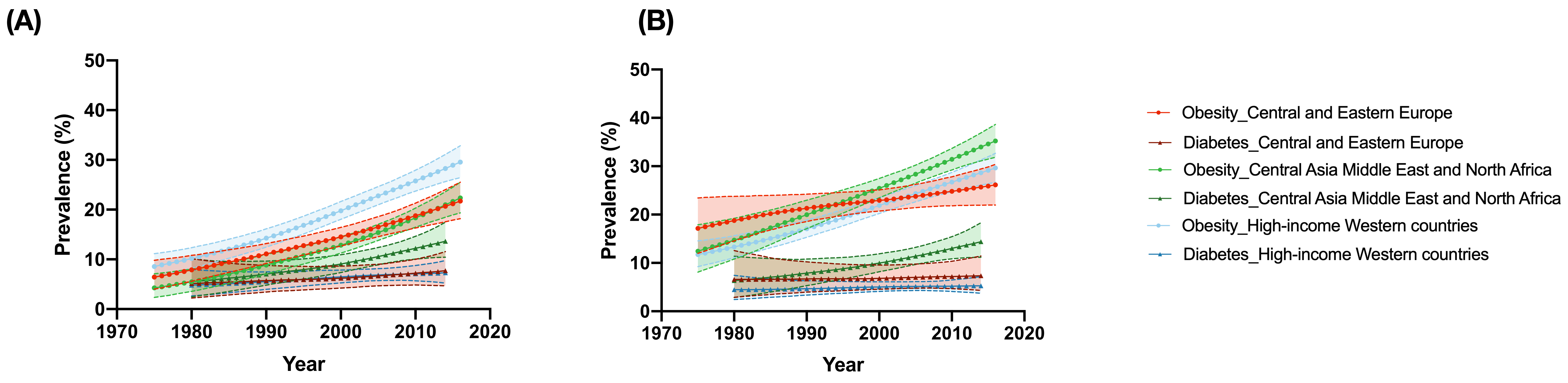

Supplementary Figure 3. Obesity and diabetes trends in Central and Eastern Europe, Central Asia Middle East and North Africa, and High-income Western countries regions, in (A) males and (B) females. Data presented is based on Non-Communicable Disease risk Collaboration (NCD-RisC) group estimates of diabetes (1975-2014) and obesity (1980-2016) across 3 regions.
