# Supplementary material for: Secondary analyses of global datasets: do obesity and physical activity explain variation in diabetes risk across populations?
Source: Int J Obes (Lond). 2021 Feb 11;45(5):944–56. doi: 10.1038/s41366-021-00764-y (PMC8081659; doi:10.1038/s41366-021-00764-y)
Supplement: Supplementary file 4 — Supplementary Figure 4 [file 41366_2021_764_MOESM4_ESM.pdf]

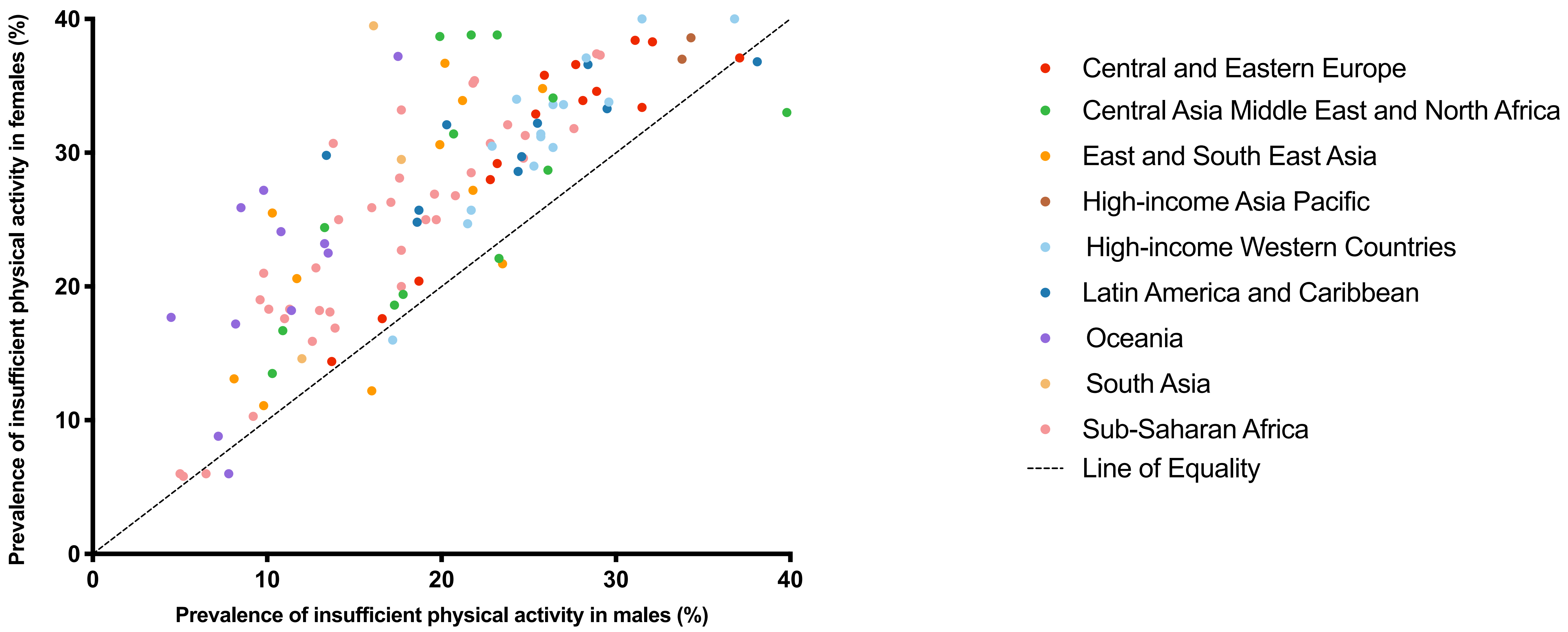

Supplementary Figure 4. Comparison of age-standardized insufficient physical activity prevalence rates in 2016 by sex. Data presented here is based on World Health Organization (WHO) estimates of insufficient physical activity rates in 2016. The scattergraph represents age-standardized insufficient physical activity prevalence rates in 168 countries, in males (x-axis) against females (y-axis). All countries were categorized into 9 super-regions and color coded in the figure. The 9 super-regions include; Central and Eastern Europe, Central Asia Middle East and North Africa, East and South East Asia, High-income Asia Pacific, High-income Western countries, Latin America and Caribbean, Oceania, South Asia, and Sub-Saharan Africa.
